# Is there scope for community health nurses to address lifestyle risk factors? the community nursing SNAP trial

**DOI:** 10.1186/1472-6955-11-4

**Published:** 2012-03-15

**Authors:** Bibiana C Chan, Rachel A Laws, Anna M Williams, Gawaine Powell Davies, Mahnaz Fanaian, Mark F Harris

**Affiliations:** 1 Centre for Primary Health Care and Equity, School of Public Health and Community Medicine, University of New South Wales, Sydney NSW 2052, Australia; 2 Prevention Research Collaboration, University of Sydney, Medical Foundation Building, 92 Parramatta Rd, Camperdown NSW 2006, Australia; 3 Illawarra Health & Medical Research Institute, Faculty of Medicine, University of Wollongong, Wollongong NSW 2522, Australia

## Abstract

**Background:**

This paper examines the opportunity and need for lifestyle interventions for patients attending generalist community nursing services in Australia. This will help determine the scope for risk factor management within community health care by generalist community nurses (GCNs).

**Methods:**

This was a quasi-experimental study conducted in four generalist community nursing services in NSW, Australia. Prior to service contacts, clients were offered a computer-assisted telephone interview to collect baseline data on socio-demographics, health conditions, smoking status, physical activity levels, alcohol consumption, height and weight, fruit and vegetable intake and 'readiness-to-change' for lifestyle risk factors.

**Results:**

804 clients participated (a response rate of 34.1%). Participants had higher rates of obesity (40.5% vs 32.1%) and higher prevalence of multiple risk factors (40.4% vs 29.5%) than in the general population. Few with a SNAPW (Smoking-Nutrition-Alcohol-Physical-Activity-Weight) risk factor had received advice or referral in the previous 3 months. The proportion of clients identified as at risk and who were open to change (i.e. contemplative, in preparation or in action phase) were 65.0% for obese/overweight; 73.8% for smokers; 48.2% for individuals with high alcohol intake; 83.5% for the physically inactive and 59.0% for those with poor nutrition.

**Conclusions:**

There was high prevalence of lifestyle risk factors. Although most were ready to change, few clients recalled having received any recent lifestyle advice. This suggests that there is considerable scope for intervention by GCNs. The results of this trial will shed light on how best to implement the lifestyle risk factor management in routine practice.

## Background

Primary health care (PHC) is an appropriate setting in which to address lifestyle risk factors because it is broadly accessible and provides continuing and comprehensive care [1]. Brief lifestyle interventions delivered in PHC have been shown to be effective for smoking cessation [2] and 'at-risk alcohol' consumption [3], and to a lesser extent for diet and physical activity [4-8].

Within PHC, family doctors are the group most often targeted for delivering lifestyle interventions. They, however, face a number of barriers, in particular a lack of time and funding [9-12]. Generalist community nurses (GCNs) in Australia are also in a good position to offer individual lifestyle intervention, because they (a) often see patients with existing chronic conditions that might benefit from lifestyle change; (b) often have ongoing contact with patients over an extended period of time; (c) mostly see clients in their own homes, and can observe the living environment and involve the wider family/carers in the intervention; and (d) may reach disadvantaged individuals with limited contact with general practice [13,14]. Our previous research has shown that GCNs see lifestyle intervention as appropriate to their role and philosophy of providing holistic care, although some thought the age of their client group limited the scope for lifestyle change [15].

Within Australia, GCNs may be either registered or enrolled nurses who are employed by the local Area Health Service. While their role varies depending upon the service in which they work, they predominately provide nursing care in people's homes, including assisting with activities of daily living, wound management, chronic disease care, continence management, palliative care, medication management, disability and dementia care. Patients can be referred following discharge from hospital, referred by their GP or other agencies or self referred.

Although community nurses are well recognised for their role in health education and promotion [16-18], few studies have evaluated their effectiveness in managing lifestyle risk factors as part of routine practice. Two overseas studies have reported positive outcomes of patients receiving advice or counselling on their smoking cessation or alcohol consumption [19,20]. However, we are not aware of any study addressing interventions across all five lifestyle risk factors in community nurses' routine practice.

This paper examines the opportunity and need for lifestyle interventions for patients attending GCN services. This will help determine the scope for risk factor management within community health care by GCNs.

## Methods

### Study Overview

This was a quasi-experimental study conducted in four generalist community nursing services in the state of NSW, Australia. This paper reports the findings of baseline data collected from clients prior to contact with the service. The study design and data collection have been described in an earlier publication [21].

### Recruitment

An expression of interest to participate in the trial was sent out to all seven Area Health Services in NSW in 2008. Four generalist community nursing services were selected on the basis of their capacity to participate and recruit sufficient numbers of clients. Clients who were referred to the participating services between September 2009 and September 2010 and met the selection criteria (Additional file 1: Appendix 1) were invited to participate in the study. Potential participants were contacted by phone on the day of referral (wherever possible) by a trained local recruitment officer.

### Data Collection

Those who consented to take part in the study were invited to participate in a computer-assisted telephone interview to collect baseline data. This included information on socio-demographic characteristics, existing health conditions, self-rated physical and mental health based on SF-12 [22], smoking status, physical activity levels [23], alcohol risk categories based on previous research [24], self-report height and weight, and intake of fruit and vegetables [25], along with readiness to change lifestyle risk factors [26] and any previous advice or referral received for existing risk factors.

### Analysis

Descriptive analyses were conducted using SPSS (version 18).

### Ethics

The project was approved by the Human Research Ethics Committees at UNSW and in each Area Health Service.

## Results

### Characteristics of clients recruited

**Figure 1:**
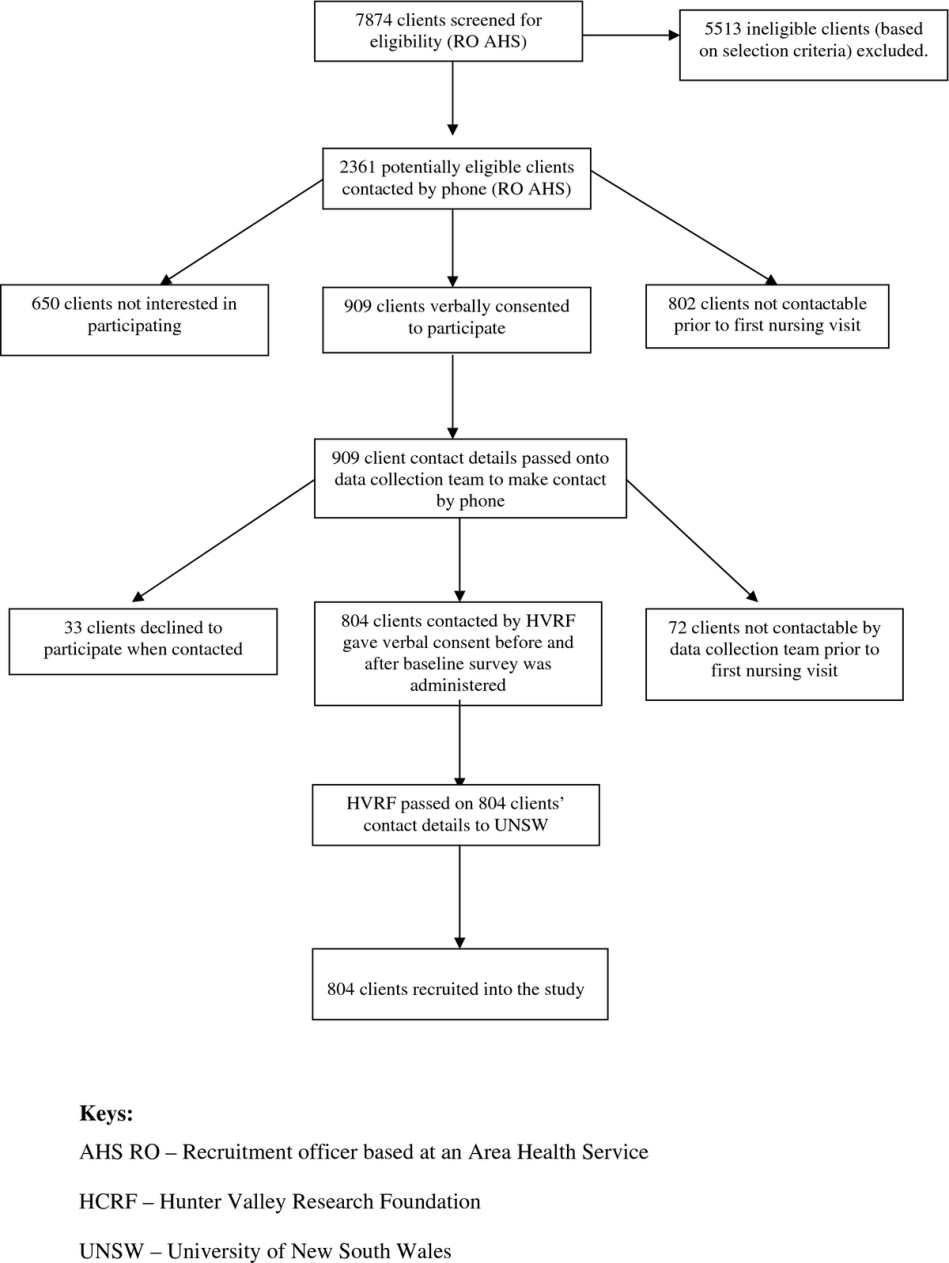
**Client recruitment process and baseline data collection**.

Between September 2009 and September 2010, 804 clients were recruited from 2361 potentially eligible clients (34.1%) (Figure 1), with similar numbers of males and females. About two-thirds (67.1%) were 60 years of age or over, and 53.1% were retired from paid work. Participants were over-represented in the middle and under-represented in the higher quintiles of the Index for Relative Socio-Economic Disadvantage [27]. Few participants spoke a language other than English or were of Aboriginal or Torres Strait Islander descent (Table 1). There were no significant differences in age and gender between those who accepted and those who declined to participate.

**Table 1:** Characteristics of CN SNAP trial clients

Characteristics	Total	
***Gender***	**N**	**%**

Female	396	49.3%

Male	408	50.7%

***Ethnicity ***		

Aboriginal/Torres Strait Islander	4	0.5%

***Language ***		

Language other than English	35	4.4%

**Employment status**		

Employed	215	26.7%

Unable to work (long-term sickness/disability)	109	13.6%

Retired from paid work	427	53.1%

Other	53	6.6%

Age (yr) *		

30-39	44	5.5%

40-49	78	9.7%

50-59	142	17.7%

60-69	256	31.9%

≥ 70	282	35.2%

**Index of Relative Socio-Economic Disadvantage **(*Postcode not known for 17 participants*)		

1^st ^quintile	148	18.8%

2^nd ^quintile	146	18.5%

3^rd ^quintile	285	36.2%

4^th ^quintile	110	14.0%

5^th ^quintile	99	12.6%

**Self-rated health status **(*How would you rate your health?*)		

Poor	308	38.3%

Good, very good or excellent	494	61.6%

**Self-rated mental health status **(*Have you felt downhearted or blue during the past month?*)		

A little or none of the time	440	54.8%
A good bit to some of the time	260	32.3%
Most to all of the time	1023	12.7%

**Health conditions**		

Hypertension (HT)	395	49.1%
Arthritis	277	34.5%
High cholesterol	239	29.7%
Cancer	213	26.5%
Other	208	25.9%
Diabetes	185	23.0%
Depression	132	16.4%
Heart disease	132	16.4%
Anxiety	105	13.16%
Asthma	104	12.9%
Respiratory	75	9.3%
Thrombosis	73	9.18%
Stroke	28	3.58%

Two participants did not disclose their age and two rated their health and mental health status as 'don't know'.

### Health status, existing conditions and risk factor profile of clients

Most patients were referred to community nursing services for wound management/dressing (74.6%); some were referred for medication administration (7.5%); general care assistance (6.5%); incontinence care (3.0%) and other (8.5%). A total of 61.6% of clients rated their own health as '*good, very good or excellent*' compared with 84.9% in the general population, perhaps reflecting the fact that 46.9% had three or more existing health conditions. In relation to mental health, 45.2% reported that during the past month they had felt *'downhearted or blue*' some or all of the time (Table 1). Almost all clients (98.0%) had at least one lifestyle risk factor and 106 (13.9%) had at least four (Table 2). There was a higher percentage of individuals with two risk factors (39.1% vs 29.5%) compared to the 65-84 year olds in the National Health Survey 2004-5 [28].

**Table 2:** Frequency of individuals with SNAPW risk factors and total number of risk factors

No. of SNAPW risk factors	N = 804 *age range: 30 - 80 yr*		National Health Survey 2004-5 ***65 -84 yr***	
no risk	9 (1.1%)		3.9%	
physical limitation otherwise no risk	8 (1.0%)			
1 risk only #	147 (18.3%)		19.4%	
2 risks #	325 (40.4%)		29.5%	
3 risks#	216 (26.9%)		27.2%	
4 risks#	90 (11.3%)		14.2%	
5 risks#	9 (1.1%)		5.8% §	

*# excluding major physical limitation; *§ 5+ risks				

Profile of risk factors			**National Health Survey 2004-5**	

	*age range: 30 - 80 yr*		*55-64 yr*	*65 -74 yr*

< 2 serves of fruit (n = 801)	336 (41.9%)		35.5%	33.5%

< 5 serves of veg (n = 796)	672 (84.4%)		80.1%	84.4%

< 7 serves of fruit & veg (n = 795)	624 (78.5%)		Not available	Not available

At risk drinking (n = 804)	297 (36.9%)		15.5%	10.4%

Smokers (n = 802)	138 (17.2%)		17.2%	10.5%

Overweight (OW) (n = 785)	263 (33.5%)		38.3%	35.8%

Obese (n = 785)	318 (40.5%)		22.1%	17.9%

OW or obese (n = 785)	581 (74.0%)		60.4%	53.7%

Unable to engage in physical activity * (n = 793)	375 (47.2%)			

Able to engage in physical activity but inadequate (n = 418)	211 (50.5%)	Sedentary	35.0%	36.3%
		
		Low exercise level	34.7%	33.0%

The 2009 National Guidelines for Alcohol Consumption is available at: http://www.health.gov.au/internet/alcohol/publishing.nsf/Content/guide-adult

The National Health Survey 2004-5 followed the old national guidelines, making direct comparisons problematic.

* those with major physical limitations which (a) limited their ability to engage in physical activity a lot and (b) estimated to last for more than 4 weeks or unsure.

### Advice and referral of clients identified with lifestyle risk factors

The majority of participants with a SNAP risk factor had not received any advice or referral in the 3 months prior to the baseline survey and very few had been provided with a referral (Table 3). The major source of advice at baseline came from the client's GP, hospital doctor or nurse, as well as family and friends. The '*Get Healthy*' phone line [29] was accessed by only two clients. There were five smokers who had used the Quit-line in the previous 3 months. About 14.4% of physically inactive clients attended local exercise programs.

**Table 3:** SNAP risk factors advice and/or referrals: Multiple responses allowed

Clients at risk	Inadequate fruit & vegetable intake (< 7 serves) (n = 625)	Inadequate physical activity (n = 211)	Smokers (n = 38)	High/risky alcohol consumption (n = 297)	Obese/over-weight with inadequate fruit or vegetable intake* (n = 580)
No advice or referral received	521(83.5%)	183 (86.7%)	104 (75.4%)	279 (93.9%)	481 (82.92%)

Received advice	85 (13.6%)	22 (10.4%)	26 (18.8%)	15 (5.1%)	86 (14.8%)

**Received referrals**	**50 (8.0%)**	**12 (5.7%)**	**19 (13.8%)**	**7 (2.4%)**	**54 (9.3%)**

Received both advice and referrals	32 (5.1%)	6 (2.8%)	11 (8.0%)	4 (1.3)	32 (5.5%)

***Source of advice***	*Multiple responses allowed*	*Multiple responses allowed*	*Multiple responses allowed*	*Multiple responses allowed*	*Multiple responses allowed*

**GP/practice nurse**	**30 (4.8%)**	**9 (4.7%)**	**19 (13.8%)**	**7 (2.4%)**	**33 (5.7%)**

Community nurse	3 (0.5%)	1 (0.5%)	1 (0.7%)	0	3 (0.5%)

**Hospital doctor/nurse**	**31 (5.0%)**	**4 (1.9%)**	**12 (8.7%)**	**5 (1.7%)**	**27 (4.7%)**

Family & friends	30 (4.8%)	9 (4.3%)	13 (9.4%)	8 (2.7%)	30 (5.2%)

Dietitian	43 (6.9%)	4 (1.9%)	0	1 (0.3%)	41 (7.1%)

Get Healthy phone-line	1 (0.2%)	1 (0.5%)	0	0	1 (0.2%)

Exercise physiologist	3 (0.5%)	1 (0.5%)	0	0	3 (0.5%)

Quitline	0	0	4 (2.9%)	0	0

Drug & alcohol counsellor	0	0	1 (0.7%)	3 (1.0%)	0

Local exercise program/gym	6(1.0%)	4 (1.9%)	0	0	7 (1.2%)

* fewer than 2 serves of fruit or fewer than 5 serves of vegetable

The proportion of patients who had risk factors and who were open to change (i.e. contemplative, in preparation or in action phase) were 65.0% of the obese or overweight, 73.8% of the smokers, 48.2% of those with at risk alcohol intake, 83.5% of those physically inactive and 59.0% of those with poor nutrition. The proportion of these who had not received previous advice and referral is shown in Table 4. This group represents those for whom advice by the GCN would be most needed. Three quarters of those at risk for low physical activity were in this category, as were approximately half of those at risk for the other risk factors.

**Table 4:** Opportunity for lifestyle intervention among clients referred to community nurse teams

Risk Factors	Number of clients identified as at risk *	Stages of Readiness to Change					Percentage of clients open to change not having received advice in last 3 months	Opportunity for intervention
	**A**	**Pre-contemplative**	**Open to change**				**C/B (%)**	**B/A × C/B** **= C/A (%)**
					
			**Contemplative**	**In preparation**	**In action**	**Percentage of 'Open to change' clients B/A (%)**		

**Overweight/obese clients to lose wt**	**574**	2015 (35.0%)	32 (5.6%)	55 (9.6%)	286 (50.2%)	**373/574 **(65.0%)	**327/373 **(87.7%)	**327/574** **(57.0%)**

**Quit smoking**	**137**	36 (26.3%)	17 (12.4%)	12 (8.8%)	72 (52.6%)	**101/137 **(73.8%)	**96/101 **(95.0%)	**96/137** **(70.1%)**

**Reduce alcohol intake**	**293**	152 (51.9%)	7 (2.4%)	28 (9.6%)	106(36.2%)	**141/293 **(48.2%)	**131/141 **(92.9%)	**131/293** **(44.7%)**

**Increase physical activity**	**208**	33 (15.9%)	28 (13.5%)	50 (24.0%)	97 (46.0%)	**175/208 **(83.5%)	**158/175 **(90.3%)	**158/208** **(76.0%)**

**Improve eating habits and nutrition**	**620**	252 (40.6%)	25 (4.0%)	69 (11.1%)	274 (44.2%)	**368/620 **(59.4%)	**318/368 **(86.4%)	**318/620** **(51.3%)**

* excluded those with missing RTC

## Discussion

This sample of individuals referred to community nurses had a high prevalence of lifestyle risk factors and associated health conditions that could benefit from lifestyle change. Participants had higher rates of obesity (40.5% compared to 32.1%), and were more likely to have multiple risk factors (40.4% compared to 29.5%) than in the population of a similar age at large [30]. Only a small proportion had received lifestyle advice or referral in the previous three months, despite the majority considering or attempting lifestyle change. The proportion of at-risk clients who were considered suitable candidates for intervention (at risk, ready or attempting change without recent advice or referral) was high for all risk factors. The absolute opportunity for intervention was highest for nutrition and physical activity, because of the higher prevalence of these risk factors in this patient group. This suggests that there is a considerable scope for GCNs to address lifestyle risk factors in these clients.

This raises the question of the most appropriate models of lifestyle intervention for community nursing clients. The community nursing SNAP trial will be testing the feasibility and effectiveness of applying the 5As model of brief lifestyle intervention within the community nursing context [21]. This consists of 1) screening clients for lifestyle risk factors as part of the routine assessment process 2) assessing readiness to change 3) providing brief advice tailored to the clients stage of change 4) referring to support services for more intensive interventions if appropriate 5) following up progress at subsequent visits. These baseline findings suggest however that intervening with this group is not going to be easy because of their age and associated co-morbidities, with almost half having three or more conditions. Participants were also more than twice as likely to report suffering from depression, compared to those in the 2007 National Survey of Mental Health and Wellbeing [31,32]. This is consistent with the finding that depression is a common co-morbidity with chronic disease conditions or multiple lifestyle risk factors [32,33].

It will be important to provide appropriate levels of support at the practitioner and service level to enable lifestyle risk factor management to be provided as part of routine practice. Our previous research with community health staff suggested that clinicians' views and perceptions can influence the extent to which they intervene to address lifestyle risk factors. We found that lifestyle risk factor management practices reflect clinician beliefs about whether they should and can address lifestyle issues. Clinician beliefs about their capacity for risk factor management reflected their views about self-efficacy, role support, and the fit between risk factor management ways of working [15,34]. It is important, therefore, to address community nurse attitudes in order to improve the delivery of lifestyle interventions to clients. It is also imperative to provide them with the necessary training (such as assess SNAPW risks and readiness to change, conduct motivational interviewing, assist clients in goal setting and offer the appropriate level of intervention) to boost their confidence in engaging in these tasks.

Many of the participants in this sample were discharged from hospital or recovering from post-surgical wounds or other illness. Their GPs might not have an opportunity to offer advice on lifestyle risk factor management yet. The process of recovery can create both opportunities and barriers for risk factor interventions. On the one hand, clients are likely to be focused on their health and on regaining normal functioning. This provides opportunities for staff at different stages of care (hospital doctors, community nurses and GPs) to support lifestyle change. On the other hand, the other demands of self-care and managing illness may overshadow lifestyle changes, and any changes that are made might not be sustained once previous health is restored.

For the minority of participants who recalled having received advice or referral, GPs and hospital doctors were the main sources of advice. GPs are often considered the 'front line' for risk factor interventions in primary health care. The incidence of recall of any recent advice from GPs was only 2.4% for alcohol consumption, 4.7% for physical activity, 4.8% for advice and 13.8% for quitting smoking. This is similar to the ranges reported from studies in general practice [35]. It suggests that there are still missed opportunities for lifestyle advice in primary medical practice. It also means that there are opportunities for GCNs to reinforce these messages at a time when patients are likely to focus more on improving their health.

Our previous research [36,37] demonstrates that lifestyle intervention by GPs is possible in general practice. However there needs to be more coordination in assisting and referring clients should more intensive intervention be required. In particular, more group based programs especially to address diet and weight may be required to achieve effective outcomes [36].

About half of the participants with poor nutrition or who were overweight/obese who recalled receiving advice had seen a dietitian. This is promising, given the evidence for effectiveness of interventions by dietitians [38], and suggests that GCNs may need to coordinate their education and advice with the dietitians whom their clients have seen, on a case-by-case basis or through in-service education. 'Family and friends' formed another important source of advice for all four SNAP risk factors. Social support can be important in initiating as well as maintaining lifestyle changes, and GCNs are well placed to encourage this through their contact with clients and their family/carers at home.

One implication for primary health care is to establish stronger links between providers and services for risk factor management to address varying needs of clients and opportunities at different stages of recovery from surgery or illness. This may need to involve GCNs (limited by the stage at which they see the patient), the general practice (limited by time, practitioners' coaching skills and communication with GCNs) and community based programs (currently limited by availability, accessibility and not being well known by GPs or GCNs). By focusing on illness prevention such as offering SNAPW risk factor lifestyle management could potentially minimise the need for medication or hospitalisation in the future.

A key challenge is that providers and services (GCNs, GPs and community based programs) have different funding models, and traditionally integration of care across these services has been poor [39]. The Australian Government recently funded Medicare Locals (NGOs) that have strong local governance, including broad community and health professional representation and have strong links to Local Hospital Networks, Local Health Districts, local communities, health professionals and service providers including GPs, allied health professionals and Aboriginal Medical Services [40]. Medicare Locals will be responsible for providing better integrated care making it easier for patients to navigate the local health care system and should be well placed to coordinate care the management of lifestyle risk factor management.

### Limitations

The data collected relied solely on clients' self-report. This could contribute to inaccurate reporting. However, the use of the computerised assisted telephone interview with software that had a built-in logic to skip questions that were not applicable and probe for more detail when warranted enhanced completion rate and minimised missing data. Social desirability may also contribute to respondents providing answers more favourable than the actual behaviours.

## Conclusion

This paper has reported the baseline prevalence of lifestyle risk factors, readiness to change and previous lifestyle advice and/or referral in clients referred to GCNs. There was a high prevalence of lifestyle risk factors, but also of readiness to change, and few clients recalled having received any recent lifestyle advice. This suggests that there is considerable scope for intervention by GCNs, but that this will need to take account of the age of clients, their state of health and the level of support they require to make changes, with coordination of care with other providers and services likely to be important. The results of this trial will shed some light on how best to implement lifestyle risk factor management in routine practice, and how far this leads to lifestyle change with this group of clients.

## Competing interests

The authors declare that they have no competing interests in the conduct of this study.

## Authors' contributions

All authors contributed to the study design, writing and reviewing the drafts, and approved the final manuscript. In particular, BC and RAL led the development of data collection tools and processes and BC conducted the statistical analysis.

## Pre-publication history

The pre-publication history for this paper can be accessed here:

http://www.biomedcentral.com/1472-6955/11/4/prepub

## Supplementary Material

Additional file 1 **Appendix 1. Selection criteria for participating clients**. Click here for file
