# Supplementary material for: Is there scope for community health nurses to address lifestyle risk factors? the community nursing SNAP trial
Source: BMC Nurs. 2012 Mar 15;11:4. doi: 10.1186/1472-6955-11-4 (PMC3337290; doi:10.1186/1472-6955-11-4)
Supplement: Additional file 1 — Appendix 1. Selection criteria for participating clients. [file 1472-6955-11-4-S1.DOCX]

**Appendix 1. Selection criteria for participating clients:**

| ***Inclusion criteria:*** |
| --- |
| - Client referred to community nursing site - Age 30-<80 years - Able to read and understand English at a level that enables client to participate in a telephone administered survey and to understand the participant information sheet. |
| **Exclusion criteria:** |
| - Palliative care client - Client receiving one-off visit or site - Client with significant cognitive impairment (unable to complete telephone administered survey). - Client for whom physical activity is contraindicated. - Client currently receiving help in changing lifestyle from a health professional (other than their GP) such as a dietitian or exercise physiologist - Client currently attending a chronic disease management program such as cardiac rehabilitation, diabetes education program etc. |
